# Outbreak investigation of acute febrile illness from the Himalayan foothills: Solving the puzzle of fever

**DOI:** 10.3389/fphar.2023.1159377

**Published:** 2023-10-26

**Authors:** Prakasini Satapathy, Kapil Goel, Vikrant Sharma, Subhabrata Sarkar, Mannat Kang, Shefali Dhingra, Ishani Bora, Kanwalpreet Kaur, Neeraj Arora, Arun Aggarwal, Radha Kanta Ratho

**Affiliations:** ^1^ Department of Virology, Postgraduate Institute of Medical Education and Research, Chandigarh, India; ^2^ Department of Community Medicine & SPH, PGIMER, Chandigarh, India; ^3^ Lab Medicine, Civil Hospital, Panchkula, India

**Keywords:** acute febrile illness, dengue fever, DENV-2, cosmopoliton genotype, outbreak

## Abstract

In September 2022, Panchkula Civil Hospital reported an outbreak of acute febrile illness (AFI) in Pinjore, located in the Himalayan foothills, Haryana, North India. There was an upsurge of fever cases. Blood samples were taken from suspected patients (*n* = 58) with AFI and subjected to serology of dengue, chikungunya, Japanese encephalitis, *leptospira* and scrub typhus. The samples were also screened for West Nile & Zika virus RNA using real-time PCR. Viral strains were characterized by sequencing. Of the 58 cases of AFI, Dengue could be identified in 45 (77.58%) followed by JE and Chikungunya in 2 cases each (3.44%), respectively. Among Dengue positive cases, 44 had monoinfection (97.77%) and 1 patient had dengue and JE. None were positive for Zika, West Nile, Scrub typhus, and *Leptospira* with the testing protocol. Four patients developed dengue with warning signs, such as abdominal pain in one patient and recurrent vomiting in the remaining three. The dengue serotype could be determined in 17 samples and revealed serotype 2. Molecular evolution analysis based on the complete envelope gene revealed that all DENV-2 strains (*n* = 13) circulated in the outbreak area belonged to the DENV-2 cosmopoliton genotype. In the early stages of infection, relying only on clinical manifestations is ineffective, so both molecular and serological assays along with clinical diagnosis are noteworthy for determining the aetiology of AFI.

## Introduction

Infection remains the common cause of febrile illness in developing countries (Mulders-Manders et al., 2015), where most primary investigations fail to ascertain a specific etiology. Thus, they are categorized into the group of AFI with a fever lasting less than 2 weeks duration. Due to limited resources in diagnosis, these illnesses remained as poorly characterized (Chao, 2012). Over the years, dengue, chikungunya, Japanese encephalitis (JE), *leptospira*, scrub typhus, and malaria are being considered as the known aetiologies of AFI cases in South Asia countries. However, in recent decades, dengue has rapidly emerged as a foremost cause of AFI in Southeast Asia (Wangdi et al., 2019). Most AFI cases can be diagnosed if the investigation is based on the clinical findings supported with laboratory investigations. Because of improper diagnosis, many a times, clinicians are forced to administer unnecessary antibiotics. Thereby leading to misuse of antimicrobials and poor patient outcomes (Long, 2016). It is crucial to determine the prevalence and epidemiology of the causative pathogens to develop protocols for therapeutic interventions of AFI.

In September 2022, Panchkula Civil Hospital reported an outbreak of fever in Pinjore, located in the Himalayan foothills of Haryana. There was an upsurge of fever cases in September 2022, (*n* = 842). Most of the patients had the acute febrile disease, body aches, and arthralgia; on investigation, the patients revealed thrombocytopenia. The present study was carried out to investigate the outbreak by characterization of the virus strains isolated from the patients.

## Methods

On the request of Haryana health authorities, the Department of Community Medicine and School of Public Health and the Department of Virology, Post Graduate Institute of Medical Research (PGIMER), Chandigarh, investigated the outbreak from 20 September to 30 September 2022. Blood samples were taken from suspected patients (*n* = 58) with AFI, serology, molecular diagnosis, and viral strain sequencing were performed at the Regional Virus Research and Diagnostic Laboratory, Department of Virology, PGIMER. Demographic and clinical details were obtained from the patients. Ethical clearance was obtained from the institutional ethics committee.

## Serological diagnosis

Blood samples were tested for Dengue NS1 antigen Elisa (Abbott, Panbio), DENV IgM capture ELISA (NIV, Pune), Chikungunya IgM ELISA (NIV, Pune), JE IgM ELISA (NIV/Pune), Scrub Typhus IgM ELISA (Detect™, Inbios) and *Leptospira* IgM ELISA (CTK). Bio Teck according to the manufacturer’s instructions. The presence of DENV IgG was detected by dengue IgG Capture ELISA (Panbio™, Abbott).

## Molecular detection

Viral RNA was extracted from serum samples using a commercial Qiagen Viral Mini Kit (Qiagen, Hiedelberg/Germany). The preparation of the first-strand cDNA was done using the high-efficiency cDNA kit (Invitrogen/United States). One step Real-Time PCR (RT-PCR) was done for the detection of dengue, chikungunya and zika viruses using CDC (Centre for Disease Control and Prevention) Trioplex RT-PCR assays (Centers for Disease Control and Prevention, 2017). Further real-time dengue PCR positive samples were subjected to serotype determination using conventional hemi-nested multiplex dengue serotype RT-PCR following the method of Lanciotti et al. (1992). The samples were also screened for West Nile virus RNA using in-house standardized real-time PCR.

Conventional PCR method was utilized for the amplification of the complete Envelope gene of DENV strains using two overlapping sets of primer pairs (Warrilow et al., 2012). The amplified E gene of representative DENV strains was purified and sanger di-deoxy sequencing was performed utilizing both forward and reverse primers. Sequences generated with forward and reverse primers were made consensus using DNASTAR Lasergene software. Phylogenetic trees based on the complete E gene coding region were constructed for the present isolates together with other reported isolates of DENV-1, DENV-2, DENV-3, and DENV-4 originating from various geographic regions. Phylogenetic trees were constructed with the help of MEGA 7.0.26 (Kumar et al., 2016). The confidence values for the branches of the phylogenetic tree were provided by bootstrap analysis of 1,000 replicates. The trees generated with several molecular algorithms were evaluated for the log-likelihood value, and the highest log-likelihood was chosen for the display.

## Results

Among the 58 recruited AFI patients selected from the outbreak location, 44.82% (26/58) patients suffered from fever. Among other clinical presentations along with fever, 13.79% (8/58), 18.96% (11/58), 10.34% (6/58) patients had myalgia, arthralgia, and skin rashes, respectively. Thrombocytopenia was detected in 12.06% (7/58) of the patients. The maximum number of cases (63.8%) was in the 19–40 years age group and 67.25% were female (Table-1). Of the 58 cases of AFI, Dengue could be identified in 45 (77.58%), followed by JE and chikungunya in 2 cases each (3.44%), respectively. Among dengue-positive cases 44 had mono-infection (97.77%) and one patient had dengue and JE. Of the 45 dengue-positive cases, 37 blood samples could be obtained within 5 days after the onset of the fever, where Dengue NS1 Ag was positive in 29 samples (78.38%), Dengue viral RNA positive in 25 samples (67.57%) and Dengue IgM antibodies in 17 samples (45.94%). However, 7 out of 8 samples (87.5%) collected after 5 days of fever had Dengue IgM antibody and NS1 Ag in 2 cases (Table-2). None were positive for Zika, West Nile, Scrub typhus, and *Leptospira* with the testing protocol. Dengue IgG was detected in 18 of the 44 (40.90%) Dengue positive patients.

**TABLE 1:** Etiological agents identified in association with socio demographic and clinical features of study participants (*N* = 58).

	Total no. of patient	DENV POS	CHKV POS	JEV POS
	N = 58	45/58 (77.58%)	2/58 (3.44%)	2/58 (3.44%)
**Gender**				
Male	19/58 (32.76%)	15/45 (33.33%)	0	0
Female	39/58 (67.24%)	30/45 (66.77)	2/2	2/2
**Age group**				
1–18 years	9/58 (15.52%)	6/45 (13.33%)	0	0
19–40 years	37/58 (63.79%)	28/45 (62.22%)	½	2/2
40–60 years	12/58 (20.68%)	11/45 (24.44%)	½	0
**Duration of Symptoms**				
≤ 5 days	45/58 (77.59%)	37/45 (82.22%)	½	½
> 5 days	13/58 (22.41%)	8/45 (17.78%)	½	½
**Clinical features**				
Fever	26/58(44.82%)	20/45(44.44%)		½
Fever with Rashes	6/58 (10.34%)	3/45 (6.66%)	½	
Fever with Myalgia	8/58(13.79%)	7/45(15.55%)		
Fever with Myalgia & Arthralgia	11/58 (18.96%)	8/45 (77.77%)	½	½
Fever with Thrombocytopenia	7/58 (12.06%)	7/45 (15.55%)	0	0

**TABLE 2:** DENV positivity spectrum according to the duration of fever.

	Total (45/58)	Patients with fever duration ≤ 5 days (37)	Patients with fever duration > 5 days (8)
DENV NS1 Ag ELISA positive	31/58 (53.4%)	29/37 (78.37%)	2/8 (25%)
DENV IgM ELISA positive	24/58 (41.3%)	17/37 (45.94%)	7/8 (87.5%)
DENV RT-PCR positive	26/58 (44.82%)	25/37 (67.56%)	1/8 (12.5%)

**FIGURE 1:**
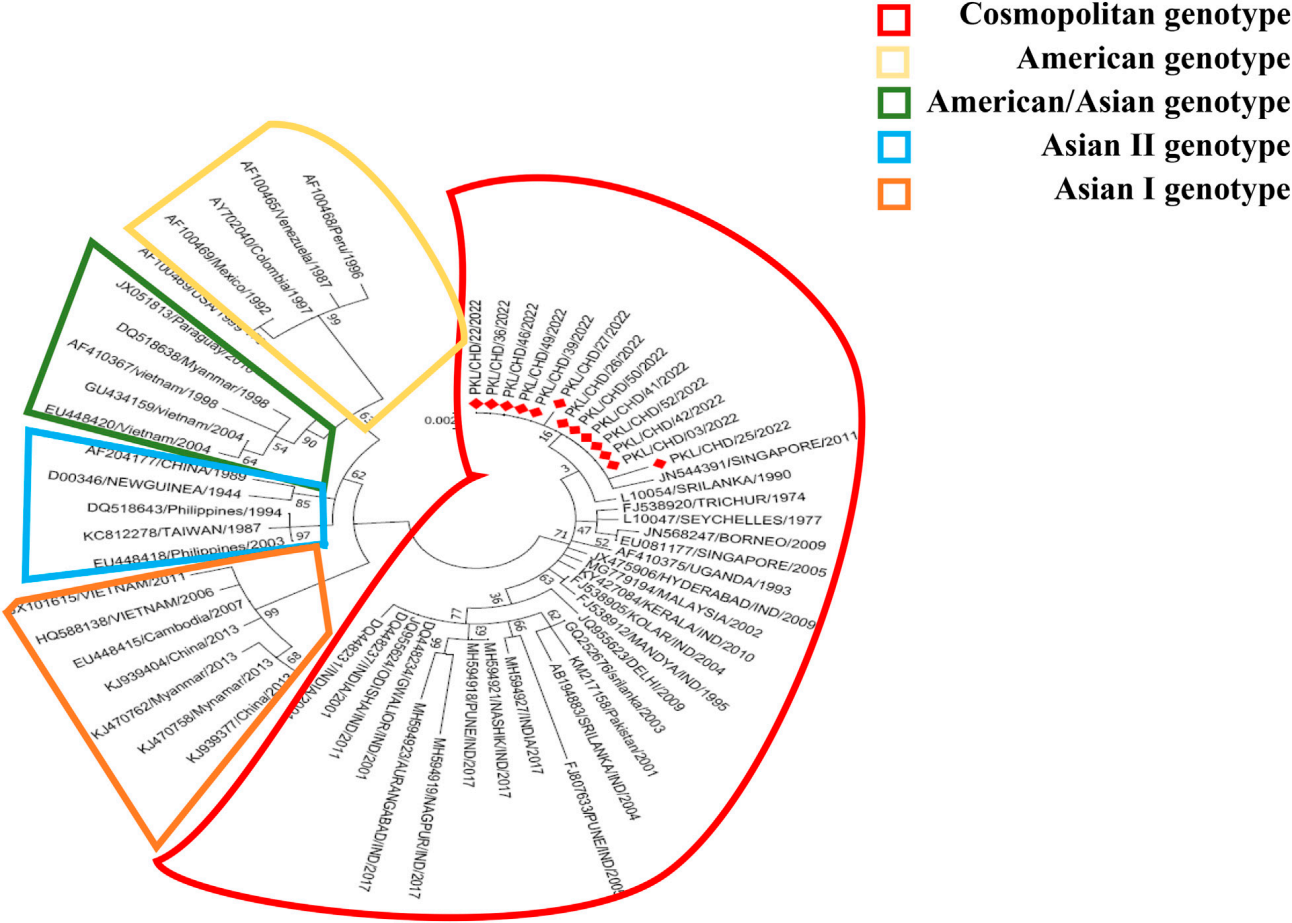
Maximum likelihood method based on the JTT matrix-based model. There were 495 amino acid positions of the 61 DENV-2 sequences in the final dataset. Evolutionary analyzes were conducted in MEGA7.

Of 26 RTPCR Dengue positives (Table 2), serotype could be determined in 17 samples, revealing serotype 2. Representative samples of DENV-2 (*n* = 13) were subjected to sequencing of the DENV-2 E gene. The aligned DNA sequences were searched with BLAST and submitted to the global gene bank with accession numbers OP808344 to OP808356. The sequences of the study strains along with the reference sequences of the DENV-2 strains belonging to the American genotype, the American/Asian genotype, Asian genotype I, Asian genotype II, and the cosmopoliton genotypes were aligned with Clustal X. To deduce the phylogenetic analysis of the study strains evolutionary analyses were performed in MEGA7. The evolutionary history was inferred using the Maximum Likelihood method based on the JTT matrix-based model. The tree with the highest logarithmic likelihood (−2,238.53) is shown in Figure 1. The percentage of trees in which the associated taxa cluster together is shown next to the branches. The initial tree(s) for the heuristic search were obtained automatically by applying the neighbour-join and BioNJ algorithms to a matrix of pairwise distances estimated using a JTT model and then selecting the topology with a superior log-likelihood value. Evolutionary analysis revealed that all DENV-2 strains (*n* = 13) circulating in the outbreak area belong to the DENV-2 cosmopoliton genotype.

## Discussion

The spread of arboviral diseases in tropical countries has become a major public health problem. The vector density increases during the post-monsoon period, thereby augmenting disease transmission. Climatic factors (temperature, rainfall, precipitation, humidity), human behavior trends, and immunity status are the main determining factors for the seasonal incidence of arboviral diseases such as dengue, chikungunya, and zika (Oki and Yamamoto, 2012; Sippy et al., 2019; Mohapatra et al., 2022). With monsoon onset, AFI cases increase and persist until winter. Scrub typhus, dengue, malaria, and chikungunya are the important etiology of AFI in India and other tropical countries (Abhilash et al., 2016; Mørch et al., 2017).

Our study confirmed that dengue is the main etiological agent (77.58%) of the fever outbreak in Panchkula, India, in the Himalayan foothills on 20 September 2022, during the post-monsoon period with an average rainfall of 426 mm. Similar observations were also found in AFI outbreak investigation studies from Arunachal Pradesh and Thailand (Khan et al., 2014; Luvira et al., 2019). Studies have reported that scrub typhus is the most prevalent among cases of AFI during the post-monsoon months (Bithu et al., 2014; Raina et al., 2018). Interestingly, in spite of the hilly area with unplanned urbanization, scrub typhus infection could not be documented in this outbreak.

In this study, 53.4% of cases were detected using the NS1 dengue antigen test, 41.3% using the IgM antibody test, while RT-PCR was positive in 44.82% (Table 2). IgG antibodies were detected in 18/44 DENV-positive cases. Samples during acute phase were positive for the NS1 antigen; however, the early appearance of IgM antibody has been reported in secondary dengue infections compared to primary dengue infection (Vazquez et al., 2010). Out of 17 cases positive for dengue IgM antibody in the acute phase of fever, 7 patients had pre-existing IgG antibodies.

Secondary infection with a different serotype or multiple infections with different serotypes may lead to severity. Of the 7 cases of fever with thrombocytopenia, all are positive for DENV. Four patients developed dengue with warning signs, such as abdominal pain in one patient and recurrent vomiting in the remaining three.

The study spanning 1994–2006 in Thailand postulated the association of DENV-2 infection with severe dengue manifestations (Fried et al., 2010). The same was corroborated by Huy et al. (2013), through a systematic review suggesting DENV-2 as a risk factor for dengue shock syndrome. Kumaria, (2010) from India documented elevated levels of liver enzymes in DENV-2 infected patients. Therefore, the sudden increase in fever cases, and high morbidity, could possibly be explained by the presence of DENV-2 strains in the locality, as revealed in the current study.

The non-sylvatic DENV-2 genotypes are classified into five genotypes (Asian I, Asian II, American, Asian-American and Cosmopoliton). The American genotype is present in Central and the South America, and Asian I and Asian II genotypes circulate on the Asian continent. Asian-American genotypes are identified in South East Asian countries as well as South American countries. The Cosmopoliton genotype is the most widespread DENV-2 found in Africa, the Middle East, and Asia-Pacific countries, including India (Weaver and Vasilakis, 2009; Yenamandra et al., 2021). The DENV-2 American genotype strains isolated from Kerala, Tamil Nadu, and New Delhi were considered the first strains circulated in 1950–1971. The Asian-American genotype was identified in Maharashtra in 2017. The cosmopolitan DENV-2 genotype was the predominant strain circulated in India from 1980 to date (Kumar et al., 2010; Kasirajan et al., 2019). Zhang et al. (2021) reported that DENV-2 cosmopoliton strains enhance pathogenicity and delay in viral clearance compared to other DENV subgenotypes. Phylogenetic tree analysis of complete envelope gene amino acids through the Maximum Likelihood method based on the JTT matrix-based model revealed that the strains of the current study belong to DENV-2 cosmopoliton genotypes, which could be the factor for a large number of fever cases and hospitalizations.

## Conclusion

Our findings demonstrate that DENV was the primary etiological agent in the fever outbreak in Panchkula, Haryana, North India. The timely diagnosis of AFI is crucial in preventing mortality and morbidity. Overlapping clinical signs, symptoms, and cross-reacting results of serological tests are the main concerns in diagnosing AFI. Serological detection accompanied by a molecular assay is helpful in the identification of sporadic outbreaks of AFI. Active syndromic surveillance and point-of-care testing should be implemented to avert AFI mismanagement. Early identification, accurate diagnosis, and early administration of proper treatment, along with enhanced global health security through appropriate control measures, would be the mainstay for controlling high-risk pathogens.

## Data Availability

The original contributions presented in the study are included in the article/supplementary material, further inquiries can be directed to the corresponding author.
